# Separation, Purification, Structural Characterization, and Anticancer Activity of a Novel Exopolysaccharide from *Mucor* sp.

**DOI:** 10.3390/molecules27072071

**Published:** 2022-03-23

**Authors:** Jiaojiao Wang, Pingchuan Yuan, Wenzhi Zhang, Chunyan Liu, Kaoshan Chen, Guodong Wang, Taili Shao

**Affiliations:** 1 Drug Research & Development Center, School of Pharmacy, Wannan Medical College, Wuhu 241002, China; wjj07080125@126.com (J.W.); 20180042@wnmc.edu.cn (P.Y.); zhangwz@ahnu.edu.cn (W.Z.); cyliu6893@126.com (C.L.); 2 Anhui Provincial Engineering Research Center for Polysaccharide Drugs, Provincial Engineering Laboratory for Screening and Re-Evaluation of Active Compounds of Herbal Medicines in Southern Anhui, Wuhu 241002, China; 3 School of Life Science, Shandong University, Jinan 250100, China

**Keywords:** exopolysaccharide from *Mucor* sp., structural characterization, anticancer activity

## Abstract

*Mucor* sp. has a wide range of applications in the food fermentation industry. In this study, a novel exopolysaccharide, labeled MSEPS, was separated from *Mucor* sp. fermentation broth through ethanol precipitation and was purified by ion-exchange chromatography, as well as gel filtration column chromatography. MSEPS was composed mostly of mannose, galactose, fucose, arabinose, and glucose with a molar ratio of 0.466:0.169:0.139:0.126:0.015 and had a molecular weight of 7.78 × 10^4^ Da. The analysis of methylation and nuclear magnetic resonance results indicated that MSEPS mainly consisted of a backbone of →3,6)-α-d-Man*p*-(1→3,6)-β-d-Gal*p*-(1→, with substitution at O-3 of →6)-α-d-Man*p*-(1→ and →6)-β-d-Gal*p*-(1→ by terminal α-l-Ara*f* residues. MTT assays showed that MSEPS was nontoxic in normal cells (HK-2 cells) and inhibited the proliferation of carcinoma cells (SGC-7901 cells). Additionally, morphological analysis and flow cytometry experiments indicated that MSEPS promoted SGC-7901 cell death via apoptosis. Therefore, MSEPS from *Mucor* sp. can be developed as a potential antitumor agent.

## 1. Introduction

*Mucor* sp., a zygomycete filamentous fungus often found in natural environments such as soils, air, fruits, and vegetables, reproduces rapidly and its hyphae develop densely [1]. Some thermotolerant species (such as *M. indicus* and *M. ramosissimus*) are obligate pathogens that affect animal and human health [2,3]. However, proteases, amylases, and lipases, produced by several *Mucor* species, including *M. circinelloides*, *M. flavus*, *M. hiemalis*, *M. mucedo*, and *M. racemosus*, hydrolyze soy components, such as proteins, carbohydrates, and fats, in the process of making classic Asian and African fermented cuisines, such as sufu, ragi, tempeh, furu, and mureha [4,5,6]. Therefore, *Mucor* sp. plays an essential role in the food fermentation industry. *Mucor* sp. has also been used in biotechnological processes to produce enzymes, particularly for the biotransformation of diverse substances, such as flavonoids, coumarins, alkaloids, and aromatic compounds, and to identify new active molecules in the pharmaceutical industry or to modify some active components for activity improvement [7,8]. Many researchers have investigated the metabolites of *Mucor* sp. as a microbial resource. Carvalho et al. reported that whole-cell lipases derived from *Mucor circinelloides* transesterify saturated short-chain fatty acids, such as lauric acid, offering a low-cost and effective alternative to lipases for industrial, biotechnological, and other applications [9]. The postharvest action of the pathogenic fungus *Aspergillus flavus* has been inhibited using chitosan, derived from *M. circinelloides* [10]. Huang et al. reported that the endophytic *M. fragilis* strain directly produces high yields of two pharmaceutically relevant bioactive chemicals utilized as anticancer and antiviral agents, podophyllotoxin and kaempferol [11].

Fungal polysaccharides, including exopolysaccharides (EPS) and endopolysaccharides, isolated from fruiting bodies, cultured mycelia, and cultured broth, exhibit a variety of biological properties, including antioxidant, antiviral, antitumor, and immunomodulatory activities [12,13,14]. An extracellular polysaccharide of *Rhizopus nigricans* inhibits the proliferation of colon cancer cells by relieving immunological inflammation in mice [15]. *Bacillus subtilis* polysaccharides inhibit A549 cell growth and promote cell apoptosis by activating the caspase-3 pathway [16], and the *Pantoea alhagi* NX-11 EPS exhibits moderate antioxidant capacity [17]. These studies indicate the immense potential of fungal polysaccharides. Additionally, numerous studies have shown that the diversity in the relative molecular weight, monosaccharide composition and content, glycosidic bond type, and sugar-ring configuration in fungal polysaccharides contributes to their structural diversity and functional novelty [18,19,20,21,22]. To date, only a few studies on the isolation, purification, structural characterization, and activity of *Mucor* fungus polysaccharides have been reported. In this study, an exopolysaccharide was isolated from *Mucor* sp. (No. CICC 3039) fermentation broth, purified, and characterized. Furthermore, its antitumor activity was studied in vitro. This study can provide a better understanding of the polysaccharides from *Mucor* sp., facilitating their medical applications to improve human health.

## 2. Results and Discussion

### 2.1. Isolation and Purification of EPS

**Figure 1:**
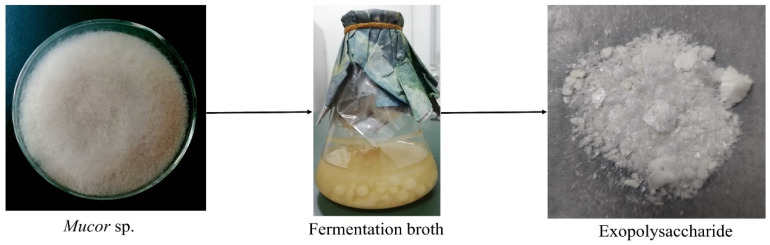
Extraction of crude exopolysaccharides from the fermentation broth.

**Figure 2:**
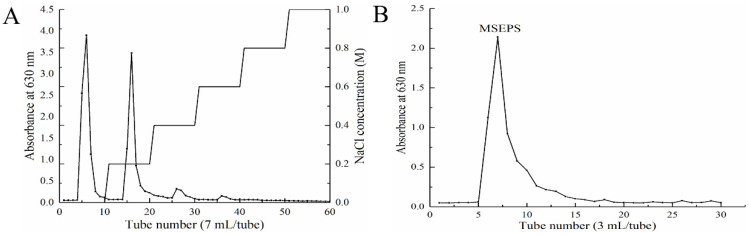
(**A**) Gradient elution diagram of the crude exopolysaccharide on the DEAE-52 ion exchange column. (**B**) Elution diagram of the anionic polysaccharide on the Sephadex G-100 gel column.

Crude EPS was obtained from *Mucor* sp. with a yield of 314 mg/L through ethanol precipitation, decolorization, deproteinization, and dialysis (Figure 1). It was first fractionated using a DEAE-52 cellulose column, and two predominant fractions were gathered (Figure 2A). A Sephadex G-100 column was used to purify the fraction eluted with a 0.2 M NaCl solution (Figure 2B). The main fractions collected were labeled MSEPS, according to the elution profile.

### 2.2. Homogeneity and Molecular Weight

**Figure 3:**
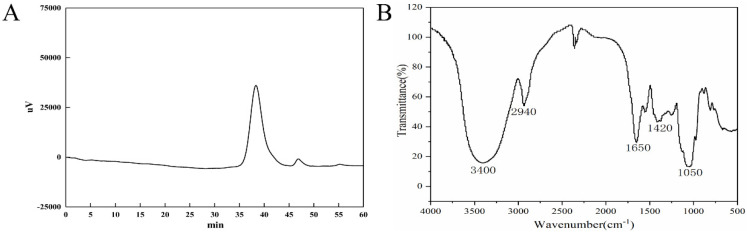
(**A**) HPGPC chromatogram of MSEPS. (**B**) Infrared spectrum of MSEPS in the range of 500–4000 cm^−1^.

The polysaccharide purity is reflected by the HPGPC spectrum. MSEPS is a homogeneous polysaccharide, as indicated by the single symmetric peak in its HPGPC spectrum (Figure 3A). The weight-average molecular weight (M_w_) of MSEPS was calculated from the standard curve to be approximately 7.78 × 10^4^ Da, its number-average molecular weight (M_n_) was 5.24 × 10^4^ Da, and its M_w_/M_n_ value was 1.48.

### 2.3. FT-IR Spectrum

The infrared spectrum of MSEPS was used to infer the characteristic functional groups in the polysaccharide (Figure 3B). The broad and strong absorption peak at 3400 cm^−1^ indicated the presence of the O–H stretching vibration, and the absorption peak at 2940 cm^−1^ represented the C–H stretching vibration [23]. Additionally, the absorption peak at 1650 cm^−1^ was due to bound water [24], and the peak at 1420 cm^−1^ was attributed to the variable angle vibration of C–H [25]. The absorption peak at 1050 cm^−1^ was caused by the stretching vibration of C–O [26].

### 2.4. Monosaccharide Composition

To identify the monosaccharide components in MSEPS, different monosaccharide standards were run on the IC system, and their retention time were recorded. According to IC analysis, MSEPS was mainly composed of mannose (Man), galactose (Gal), fucose (Fuc), arabinose (Ara), and glucose (Glc) in a molar ratio of 0.466:0.169:0.139:0.126:0.015, with trace amounts of glucuronic acid and galacturonic acid (Table 1). The monosaccharides Man, Gal, and Ara are frequently found in other fungal polysaccharides [27,28]. Previous studies have shown that the types and proportions of monosaccharides depend on culture conditions, methods of isolation and purification, and the polysaccharide source [29,30].

### 2.5. Methylation Analysis

The location of linkages between monosaccharide residues in polysaccharides can be determined through methylation analysis. As summarized in Table 2, the main linkages of monosaccharides were →6)-Man*p*-(1→, →3,6)-Man*p*-(1→, →6)-Gal*p*-(1→, →3,6)-Gal*p*-(1→, Fuc*p*-(1→, and Ara*f*-(1→. The linkage pattern →3,6)-Man*p*-(1→ and →3,6)-Gal*p*-(1→ indicated that MSEPS is a branched polysaccharide.

### 2.6. NMR Spectroscopy

**Figure 4:**
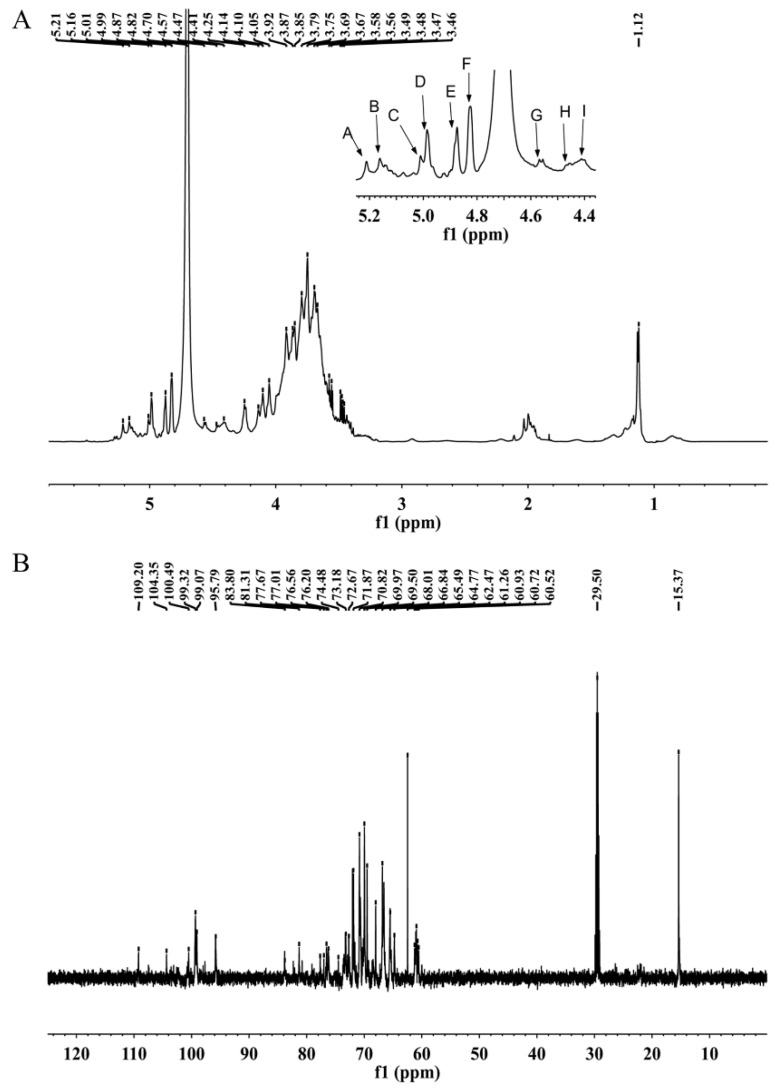
One-dimensional NMR spectra of MSEPS: (**A**) ^1^H-NMR spectrum; (**B**) ^13^C-NMR spectrum.

The precise structural features of MSEPS were further elucidated by ^1^H, ^13^C, ^1^H–^1^H correlated spectroscopy (COSY), heteronuclear single-quantum correlation spectroscopy (HSQC), heteronuclear multiple bond correlation (HMBC), and nuclear overhauser effect spectroscopy (NOESY) NMR spectroscopy. In the ^1^H-NMR spectrum of MSEPS (Figure 4A), the peaks at δ 5.21, 5.16, 5.01, 4.99, 4.87, 4.82, 4.57, 4.47, and 4.41 ppm correspond to anomeric protons, which were labeled residues A, B, C, D, E, F, G, H, and I, respectively. The peak at δ 1.12 ppm was assigned to the proton signal of the methyl group in fucose residues. Usually, signals located in the δ 4.8–5.5 ppm region are due to anomeric protons in α-configuration pyranose units. By contrast, the anomeric proton chemical shifts from δ 4.4–4.8 ppm are due to the β-configuration pyranose units [31]. Therefore, the polysaccharide MSEPS contains both α- and β-pyranose structures. The other signals at δ 3.2–4.3 ppm were attributed to the sugar-ring protons. In the ^13^C spectrum of MSEPS (Figure 4B), the signals were mainly distributed in the δ 60–110 ppm range. Six anomeric carbon signal peaks were detected at δ 109.20, 104.35, 100.49, 99.32, 99.07, and 95.79 ppm. The chemical shifts of the non-substituted C-6 occurred at δ 60–64 ppm, which shifted to δ 65–70 ppm upon substitution at C-6. The signals for C-2, C-3, and C-6 on the sugar ring appeared at δ 70–77 ppm and were shifted to the δ 78–85 ppm range upon substitution [32,33]. The carbon signal at δ 15.37 ppm was attributed to the methyl group in fucose residues.

**Figure 5:**
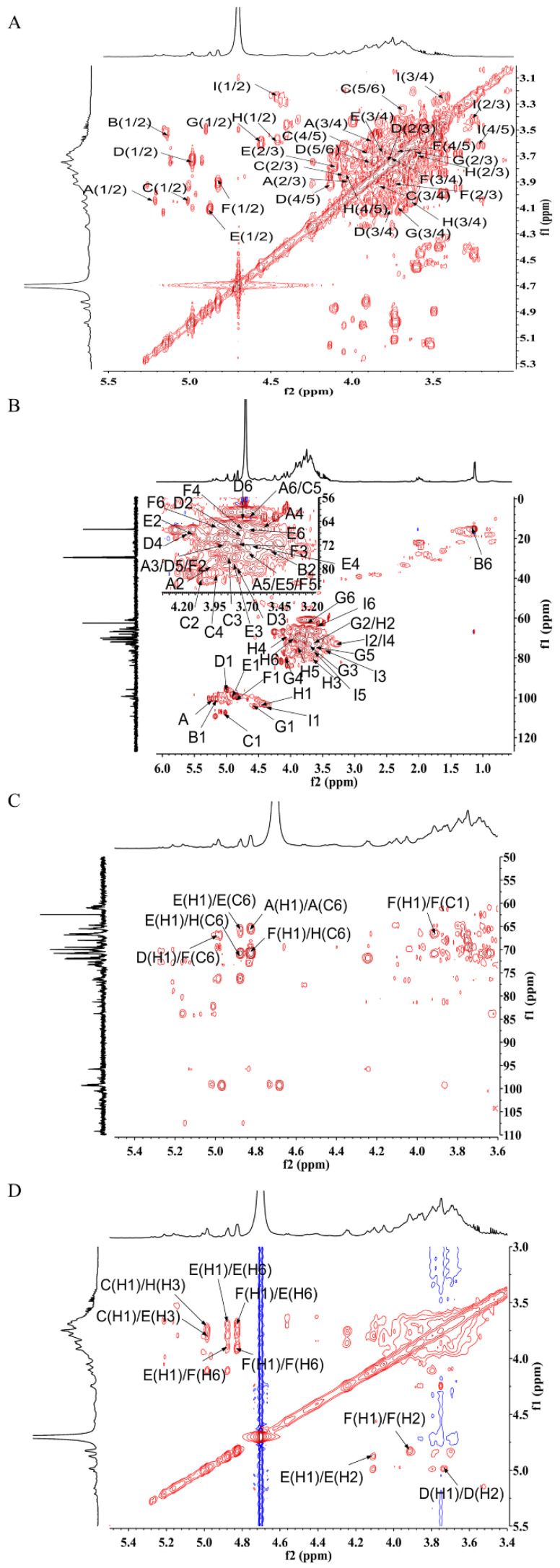
Two-dimensional NMR spectra of MSEPS: (**A**) ^1^H–^1^H COSY spectrum; (**B**) ^1^H–^13^C HSQC spectrum; (**C**) ^1^H–^13^C HMBC spectrum; (**D**) NOESY spectrum.

The signals of the corresponding hydrogens on adjacent carbons were reflected by the ^1^H–^1^H COSY spectrum. C–H correlations on the same carbon were obtained from the HSQC spectrum. Residue A was identified by the correlation peak at H-1/C-1 5.21/101.75 ppm from the HSQC spectrum. In the ^1^H–^1^H COSY spectrum (Figure 5A), hydrogen signals at δ 5.21, 4.00, 3.88, 3.60, 3.70, and 3.69 ppm were attributed to the presence of H-1, H-2, H-3, H-4, H-5, and H-6a in residue A, respectively. The relevant carbon signals from C-1 to C-6 were observed at δ 101.75, 80.21, 71.32, 68.19, 74.50, and 62.30 ppm, respectively, in the HSQC spectrum (Figure 5B). The carbon signal at δ 80.21 ppm was attributed to the C-2 signal of the (1→2)-linked mannose. By comparison with literature, it could be inferred that residue A was →2)-α-d-Man*p*-(1→ [34]. For residue C, proton signals at δ 5.01, 4.13, 3.83, 3.91, 3.69, and 3.36 ppm were related to the corresponding carbon signals at δ 108.77, 82.70, 77.80, 85.10, and 62.33 ppm, respectively. The chemical shift of the heterotopic carbon of residue α-l-Ara*f* was above δ 108 ppm. Therefore, residue C was deduced to be α-l-Ara*f*-(1→, due to the chemical shifts of C-1 [35]. The chemical shifts of C-1 and C-3 in residue D were δ 95.78 and δ 80.50 ppm, respectively, which were shifted toward lower fields on substitution. Therefore, it could be inferred that residue D was →3)-α-d-Man*p*-(1→. Similarly, cross-signals were observed at δ 4.87/100.84, 4.09/68.18, 3.82/79.46, 3.65/72.70, 3.73/74.64, and 3.68/66.91 ppm, which were assigned to H-1/C-1, H-2/C-2, H-3/C-3, H-4/C-4, H-5/C-5, and H-6a/C-6 in residue E, respectively. The chemical shifts of C-1 and C-3 were significantly shifted to lower fields compared to the chemical shifts of the unsubstituted sugar ring. Therefore, residue E was considered to be →3,6)-α-d-Man*p*-(1→. The cross signals at δ 4.82/100.80, 3.91/71.37, 3.75/71.87, 3.75/67.89, 3.68/74.50, and 3.92/66.82 ppm originated from H-1/C-1, H-2/C-2, H-3/C-3, H-4/C-4, H-5/C-5, and H-6a/C-6 in residue F, respectively. The chemical shift of C-6 was shifted toward a lower field (δ 66.82 ppm) compared to that of the unsubstituted C-6 (in the δ 60–64 ppm range). Therefore, residue F was considered to be →6)-α-d-Man*p*-(1→ [36]. The chemical shift of the heterotopic carbon of residue G was δ 105.80 ppm. According to the literature, the chemical shift of the heterotopic carbon of residue β-d-Gal*p* lies in the δ 103–106 ppm range. Additionally, the C-4 position of residue G was replaced, as indicated by movement of the chemical shift of C-4 toward a lower field, to δ 79.66 ppm. Therefore, residue G was confirmed to be →4)-β-d-Gal*p*-(1→ [37]. The chemical shifts from C-1 to C-6 of residue H were acquired from the HSQC spectrum on the basis of the C–H pairs. Residue H was postulated to contain the β-d-Gal*p* fragments because the chemical shift of the heterotopic carbon was δ 104.69 ppm. The downfield shifts of C-3 (δ 81.50 ppm) and C-6 (δ 70.76 ppm) confirmed residue H to be →3,6)-β-d-Gal*p*-(1→ [38]. Similarly, residue I was considered to be β-d-Gal*p*-(1→. In residue B, the hydrogen signal at δ 1.14 ppm and the carbon signal at δ 15.31 ppm corresponded to the methyl group in fucose. Combining this information with results of the methylation analysis, residue B was confirmed to be α-l-Fuc*p*-(1→ [39]. The chemical shifts of carbon and hydrogen for all residues are listed in Table 3.

The sequences of the residues in MSEPS were determined from HMBC and NOESY spectra. As shown in the HMBC spectrum (Figure 5C), the anomeric proton of residue F had a strong cross-peak with its own C-6, indicating the presence of →6)-α-d-Man*p*-(1→6)-α-d-Man*p*-(1→. The anomeric proton of residue F also showed a strong inter-residual cross peak with the C-6 of residue H, indicating →6)-α-d-Man*p*-(1→3,6)-β-d-Gal*p*-(1→ linkage. Meanwhile, the anomeric proton of residue E had a self-correlation with its C-6, indicating the existence of →3,6)-α-d-Man*p*-(1→3,6)-α-d-Man*p*. Additionally, the anomeric proton of residue E showed strong coupling with the C-6 of residue H, indicating →3,6)-α-d-Man*p*-(1→3,6)-β-d-Gal*p*-(1→ linkage. Coupling between the anomeric proton of residue D and C-6 of residue F indicated the existence of →3)-α-d-Man*p*-(1→6)-α-d-Man*p*-(1→. The NOESY spectrum (Figure 5D) further confirmed the results obtained from the HMBC spectrum analysis. The anomeric proton of residue C was associated with the H-3 of residue E and the H-3 of residue H, indicating the linkage of α-l-Ara*f*-(1→ to the O-3 of →6)-α-d-Man*p*-(1→, and →6)-β-d-Gal*p*-(1→.

**Figure 6:**
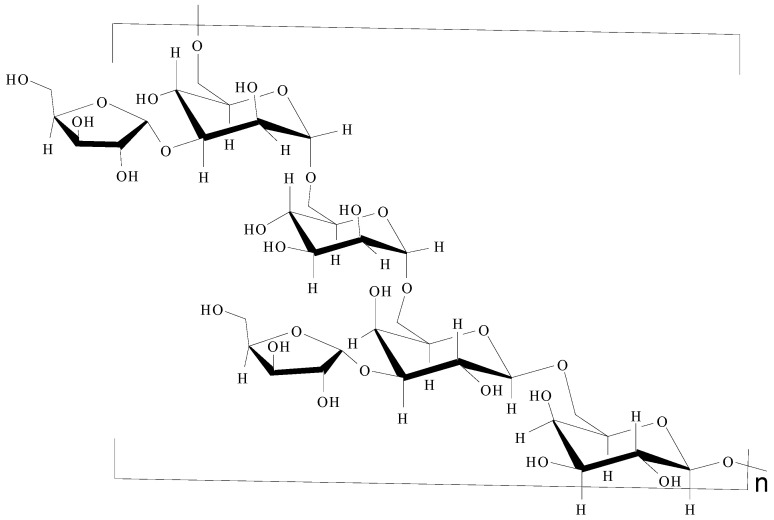
One of the possible repeating structures of MSEPS.

On the basis of the above analysis, a putative structure of MSEPS was established. The main skeleton of MSEPS consists of →3,6)-α-d-Man*p*-(1→3,6)-β-d-Gal*p*-(1→ residues, with terminal α-l-Ara*f* attached to the main chain at O-3 of →6)-α-d-Man*p*-(1→, and →6)-β-d-Gal*p*-(1→. One of the possible repeating structures of MSEPS is shown in Figure 6.

### 2.7. Anticancer Activity of MSEPS

#### 2.7.1. Effect of MSEPS on SGC-7901 Cell Inhibition

**Figure 7:**
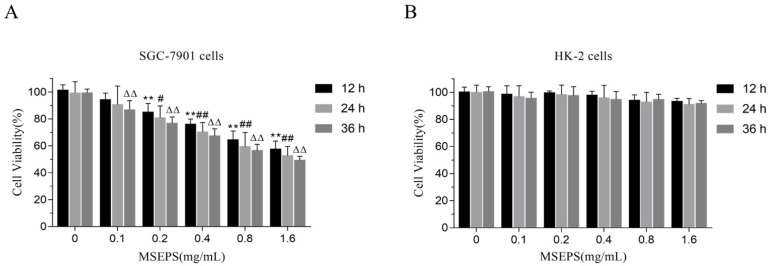
(**A**,**B**) Effects of MSEPS treatment on the viability of SGC-7901 and HK-2 cells. Cells were treated with different concentrations of MSEPS for 12, 24, or 36 h, and the cell viability was detected by MTT assay. Each value is presented as the mean ± SD (*n* = 5). ** *p* < 0.01 compared to the control group (12 h); ^#^ *p* < 0.05, ^##^ *p* < 0.01 compared to the control group (24 h); ^ΔΔ^ *p* < 0.01 compared to the control group (36 h).

The MTT method was used to investigate the in vitro anticancer activity of MSEPS, with HK-2 cells as control. The inhibitory effect of MSEPS on SGC-7901 cell growth became more pronounced with increasing concentrations (Figure 7A). At the highest dose of 1.6 mg/mL, the inhibition rate of SGC-7901 cells for 12 h, 24 h, or 36 h was 42.47%, 47.21%, and 50.65%, respectively. Moreover, the same concentration of MSEPS increased the inhibition rate of SGC-7901 cells at longer treatment times. By contrast, the MSEPS treatment did not significantly affect the viability of HK-2 cells, even after treatment for 36 h (Figure 7B). Additionally, the viabilities of the SGC-7901 and HK-2 cells were significantly different for treatments with MSEPS concentrations higher than 0.2 mg/mL. Therefore, MSEPS exhibited low toxicity against non-tumor cells, while inhibiting SGC-7901 cell proliferation in a concentration- and time-dependent manner.

#### 2.7.2. Effect of MSEPS on Morphological Changes of SGC-7901 Cells

**Figure 8:**
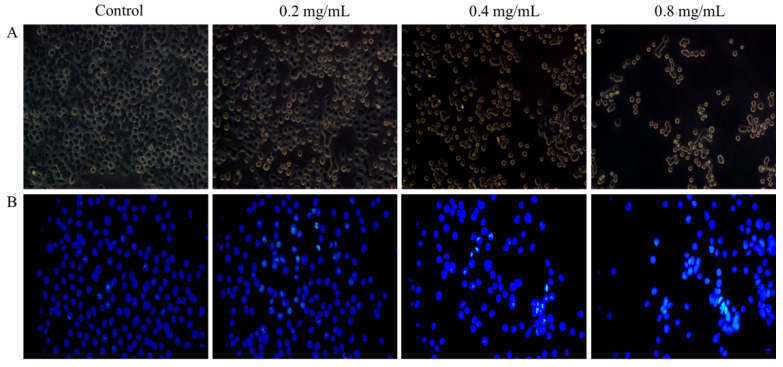
Effect of MSEPS treatment on the cells, or nuclear morphological changes of SGC-7901 cells. SGC-7901 cells were treated with different concentrations of MSEPS for 24 h: (**A**) cells observed microscopically (200×); (**B**) cells dyed with Hoechst 33,258 and visualized with fluorescence microscopy (400×).

The morphological changes of SGC-7901 cells, after treatment with different concentrations of MSEPS for 24 h, were observed under an inverted microscope. As shown in Figure 8A, MSEPS-treated SGC-7901 cells underwent a series of morphological changes, including cell contraction and rounding, arrangement loosening, and a significant decline in the number of attached cells, while the untreated SGC-7901 cells exhibited normal morphology. To investigate the link between the antiproliferative activity of MSEPS and apoptosis induction, the nuclear morphology of SGC-7901 cells was examined after Hoechst 33,258 (nuclear-specific fluorescent dye) staining. As shown in Figure 8B, the untreated cells exhibited evenly distributed nuclei with regular oval shapes, whereas the characteristic apoptotic signs, including karyopyknosis, chromatin compaction, nuclear fragmentation, and bright blue fluorescence were observed upon treating the cells with increasing concentrations of MSEPS [40]. Therefore, MSEPS can trigger apoptosis, contributing to the inhibition of SGC-7901 cell growth.

#### 2.7.3. Effect of MSEPS on the Apoptotic Induction of SGC-7901 Cells

**Figure 9:**
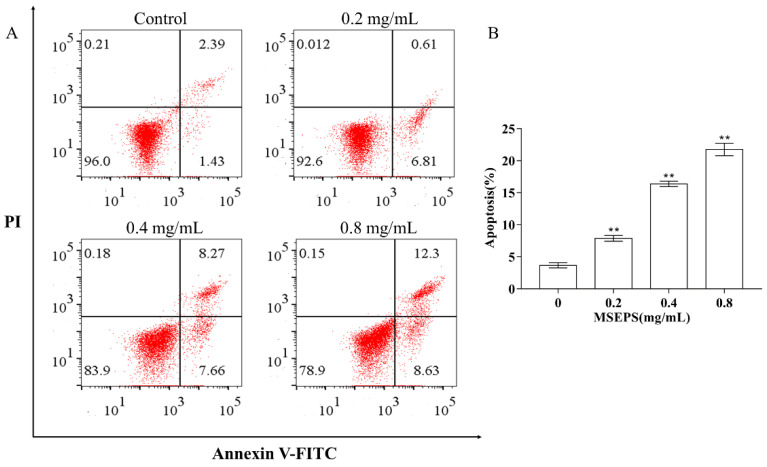
(**A**,**B**) Effects of MSEPS treatment on apoptosis of SGC-7901 cells. Representative dot plots of annexin V/PI staining detected by flow cytometry after treatment with different concentrations of MSEPS for 24 h. Each value represents the mean ± SD (*n* = 3). ** *p* < 0.01 compared to the control group.

Annexin V–FITC/PI staining, combined with flow cytometry, confirmed the apoptotic effects of MSEPS on SGC-7901 cells. As shown in Figure 9, early and late apoptotic cells, as well as normal cells, were detected after 24 h treatment with various concentrations of MSEPS. The exposure of SGC-7901 cells to MSEPS increased the ratios of total apoptotic cells to 7.42%, 15.93%, and 20.93%, in a dose-dependent manner. The untreated group exhibited high cell viability, with 3.82% total apoptosis. Therefore, the growth inhibition of SGC-7901 cells was related to the apoptosis-induction effect.

## 3. Materials and Methods

### 3.1. Materials and Chemicals

The China Center of Industrial Culture Collection provided the *Mucor* sp. strain (CICC 3039) used in this study. Cellulose DE-52 and Sephadex G-100 chromatography resins were obtained from Beijing Solibao Technology Co., Ltd. (Beijing, China). Dextran standards (668, 410, 273, 148, 81, 49, 24, 12, and 5 kDa) and monosaccharide standards (fucose, rhamnose, arabinose, galactose, glucose, xylose, mannose, fructose, ribose, galacturonic acid, guluronic acid, glucuronic acid, and mannuronic acid) were acquired from Sigma-Aldrich Co., Ltd. (St. Louis, MO, USA). DMEM medium, RPMI-1640 medium, fetal bovine serum (FBS), penicillin, and streptomycin were purchased from GIBCO GRL (USA). Hoechst 33,258, 3-(4,5-dimethylthiazol-2-yl)-2,5-diphenyltetrazolium (MTT), and annexin V–FITC apoptosis kits were supplied by the Beyotime Institute of Biotechnology (Nanjing, China). Chromatographic-grade reagents were used in high-performance gel permeation chromatography (HPGPC) and gas chromatography–mass spectrometry (GC-MS). All other chemicals used were of analytical grade.

### 3.2. Isolation and Purification of EPS from Mucor sp.

*Mucor* sp. was cultivated in a potato dextrose broth for 6 days at 28 °C and 130 rpm. The fermented broth was centrifuged for 10 min at 5000 rpm. Four volumes of 95% ethanol were added to the resulting supernatant and kept overnight at 4 °C. The precipitate obtained by centrifugation at 8000 rpm for 10 min was subsequently redissolved in deionized water. The precipitate was removed by centrifugation at 8000 rpm for 10 min. The Sevag reagent was used to eliminate the protein from the supernatant [41], followed by the decolorization of the aqueous phase by a macroporous adsorption resin (D301), dialysis, and lyophilization.

The lyophilized powder was prepared as a 10 mg/mL solution and injected into a DEAE-52 column (2.5 × 40 cm) for elution using 0, 0.2, 0.4, 0.8, and 1 M NaCl solutions at 0.7 mL/min flow rate. After collection by an automatic collector, the eluate was analyzed using the anthrone–sulfuric acid method, and an elution curve was plotted, considering tube numbers and absorbance values [42]. The fraction eluted with 0.2 M NaCl was further purified using a Sephadex G-100 column (1.5 × 60 cm), at a 0.3 mL/min flow rate, with ultrapure water. The main fractions were collected according to the elution curve, and the purified polysaccharide, labeled MSEPS, was obtained after dialysis and lyophilization.

### 3.3. Characterization

#### 3.3.1. Homogeneity and Molecular Weight of MSEPS

An HPGPC instrument, equipped with an Agilent LC-10A platform, a BRT105-104-102 chromatography column (8 × 300 mm, Borui Saccharide, Biotech. Co., Ltd., Yangzhou, China), and a refractive index detector (RID-10A, Shimadzu, Kyoto, Japan), was effectively used to determine the homogeneity and molecular weight of MSEPS. The detection conditions were as follows: 40 °C column temperature, 0.05 M NaCl mobile phase, and 0.6 mL/min flow rate. MSEPS solution (20 μL, 5 mg/mL) was injected into the column, and the linear curve was plotted using different molecular weights of dextran (668, 410, 273, 148, 81, 49, 24, 12, and 5 kDa).

#### 3.3.2. Fourier-Transform Infrared (FT-IR) Spectroscopy Analysis

The infrared spectrograms of MSEPS were acquired using an FTIS-8400S spectrometer (Shimadzu, Kyoto, Japan). A mixture of the dried sample (2 mg) and KBr (200 mg) was pressed into a pellet, and FT-IR spectra were recorded in the 4000–500 cm^−1^ frequency range [43].

#### 3.3.3. Monosaccharide Composition Analysis

Analysis of monosaccharide composition was performed on a high-performance ion exchange chromatography (HPIC) system, equipped with a Dionex™ Carbopac™ PA20 column (ICS5000, 3 × 150 mm, ThermoFisher, MA, USA) [44]. MSEPS was hydrolyzed by 10 mL of 3 M trifluoroacetic acid (TFA) at 120 °C for 3 h. The MSEPS hydrolysates were dried with nitrogen to remove TFA. The obtained sample was dissolved in methanol and dried thrice for the complete removal of residual TFA. Subsequently, the dried hydrolysates were reconstituted in ultrapure water and injected into the HPIC system using water, 15 mM NaOH, and 15 mM NaOH/100 mM NaOAc as the mobile phase at a flow rate of 0.3 mL/min. The injection volume was 5 μL, and the column temperature was kept at 30 °C.

#### 3.3.4. Methylation Analysis

MSEPS was analyzed for methylation by GC–MS using a previously published approach [45]. MSEPS (3 mg) was ultrasonically dissolved in dimethyl sulfoxide (DMSO, 1.0 mL), followed by addition of sodium hydroxide (0.6 mL) in a nitrogen flow. Subsequently, cold CH_3_I (1 mL) was added to the mixture, which was then ultrasonically reacted for 1 h at 30 °C. The addition of ultrapure water (2 mL) terminated the methylation reaction. The methylation product was hydrolyzed by of 2 M TFA (1 mL) at 120 °C for 2 h. Thereafter, the hydrolysis product was dissolved in water and reduced with NABH_4_ for 8 h. Acetic anhydride was added to the dried sample and heated at 100 °C for 2 h in a closed chamber. The final acetylation product was dissolved in chloroform (3 mL), and the chloroform layer was washed with water. Shimadzu GC–MS (Shimadzu, Kyoto, Japan), with an RXI-5 SIL MS capillary column (30 m × 0.25 mm × 0.25 m), was used to analyze the methylation of alditol acetates. The temperature was set to rise from 120 to 250 °C at a rate of 3 °C/min, and then maintain 250 °C for 5 min. The temperature of the detector and injector were both set at 250 °C.

#### 3.3.5. Nuclear Magnetic Resonance Spectroscopy (NMR) Analysis

MSEPS (50 mg) was freeze-dried after being dissolved in 0.5 mL D_2_O. This process was carried out thrice for the full replacement of hydrogen, followed by redissolution of the samples in 0.5 mL D_2_O. A Bruker AVANCE 600 MHz spectrometer (Bruker, Karlsruhe, Germany) was used to record the NMR spectra at 25 °C [46].

### 3.4. In Vitro Anticancer Activities

#### 3.4.1. Cell Lines and Culture

Human gastric adenocarcinoma (SGC-7901) cells were purchased from the Beyotime Institute of Biotechnology (Nanjing, China), and human renal tubular epithelial (HK-2) cells were used as controls. The SGC-7901 and HK-2 cells were cultured in an incubator at 37 °C with 5% CO_2_, using RPMI 1640 and DMEM complete medium containing 10% FBS, respectively.

#### 3.4.2. Measurement of Cell Viability

The viability of SGC-7901 and HK-2 cells were assessed by the MTT method, as reported previously [47]. The SGC-7901 and HK-2 cell suspensions were inoculated at a density of 5 × 10^3^ cells/mL into a 96-well plate and cultured for 24 h. MSEPS solutions, at final concentrations of 0, 0.1, 0.2, 0.4, 0.8, and 1.6 mg/mL, were added to the wells and incubated for 12 h, 24 h, or 36 h. Subsequently, 20 μL MTT (5 mg/mL) was added to each well and incubated for 4 h. The supernatants from the wells were carefully aspirated, followed by the addition of DMSO (150 µL) to each well for crystal dissolution. Absorbance values for each well were measured at 490 nm using a full-wavelength microplate reader. All measurements were performed thrice. Cell viability rates were calculated as follows: cell viability rate (%) = OD_sample_/OD_control_ × 100%, where OD_control_ and OD_sample_ are absorbances of the untreated cells and MSEPS-treated cells, respectively.

#### 3.4.3. Morphologic Observations

SGC-7901 suspensions were inoculated at a density of 2 × 10^5^ cells/mL into a six-well plate and cultured for 24 h. Cells were treated with various doses of MSEPS (0, 0.2, 0.4, and 0.8 mg/mL) for 24 h. Changes in cell morphology were observed using an inverted microscope (Olympus, Tokyo, Japan).

#### 3.4.4. Hoechst 33,258 Staining

SGC-7901 suspensions were inoculated at a density of 1 × 10^4^ cells/mL into a 24-well plate and cultured for 24 h. After another 24 h of exposure to varied concentrations of MSEPS (0, 0.2, 0.4, and 0.8 mg/mL), cells were fixed in 4% paraformaldehyde for 15 min and then stained with a nuclear dye (Hoechst 33,258) for 20 min, under light-proof conditions. The fixed cells were washed twice with PBS and examined under an inverted fluorescence microscope (Olympus, Tokyo, Japan) to observe changes in the nuclei.

#### 3.4.5. Apoptosis Measurement

An FITC-labeled annexin V probe was used to detect early apoptosis in cells. SGC-7901 suspensions were inoculated at a density of 2 × 10^5^ cells/mL into a six-well plate and cultured for 24 h. After being subjected to different concentrations of MSEPS (0, 0.2, 0.4, and 0.8 mg/mL) for 24 h, the cells were collected by centrifugation, after digestion with EDTA-free trypsin. The collected cells were resuspended in a binding solution (500 µL), followed by the addition of Annexin V–FITC (5 μL) and propidium iodide (5 μL). The samples were kept under light-proof conditions for 20 min, before being analyzed using a flow cytometer (FACSVerse, Becton Dickinson, Franklin Lake, NJ, USA).

### 3.5. Statistical Analysis

For data collection, all experiments were repeated at least twice. The results were presented as mean ± SD and statistically analyzed using the Student’s *t*-test and one-way analysis of variance (ANOVA). A *p*-value < 0.05 was considered to be statistically significant.

## 4. Conclusions

A novel heteropolysaccharide named MSEPS, with an M_w_ of 7.78 × 10^4^ Da, was obtained from a fermentation broth of *Mucor* sp. The chemical structure of MSEPS was elucidated by monosaccharide composition, methylation, and NMR spectroscopic analysis. Additionally, MTT assays, morphological observations, and flow cytometry analyses indicated that MSEPS could selectively inhibit SGC-7901 cell growth by inducing apoptosis. This study indicated that MSEPS could be a promising anticancer agent against human gastric cancer cells and could facilitate future research on the influence of *Mucor* sp. metabolites on human health. Future research may investigate the mechanism of apoptosis induction in SGC-7901 cells to confirm the effects of MSEPS on human gastric cancer cells.

## Figures and Tables

**Table 1:** Monosaccharide composition analysis of MSEPS.

Retention Time (min)	Monosaccharide Composition	Relative Molar Ratio
5.684	fucose	0.139
11.359	rhamnose	0
11.909	arabinose	0.126
14.900	galactose	0.169
16.967	glucose	0.015
20.209	xylose	0
20.750	mannose	0.466
24.367	fructose	0
27.884	ribose	0
44.942	galacturonic acid	0.005
45.992	guluronic acid	0
48.034	glucuronic acid	0.008
50.817	mannuronic acid	0

**Table 2:** Methylation analysis data of MSEPS.

RT	Methylated Sugar	Mass Fragments (*m*/*z*)	Molar Ratios	Type of Linkage
9.223	2,3,5-Me_3_-Ara*f*	43,71,87,101,117,129,145,161	0.119	Ara*f*-(1→
11.669	2,3,4-Me_3_-Fuc*p*	43,59,72,89,101,115,117,131,175	0.128	Fuc*p*-(1→
16.274	2,3,4,6-Me_4_-Man*p*	43,71,87,101,117,129,145,161,205	0.025	Man*p*-(1→
17.271	2,3,4,6-Me_4_-Gal*p*	43,71,87,101,117,129,145,161,205	0.018	Gal*p*-(1→
20.564	3,4,6-Me_3_-Man*p*	43,87,129,161,189	0.041	→2)-Man*p*-(1→
20.885	2,3,6-Me_3_-Gal*p*	43,87,99,101,113,117,129,131,161,173,233	0.025	→4)-Gal*p*-(1→
22.241	2,3,4-Me_3_-Glc*p*	43,87,99,101,117,129,161,189,233	0.009	→6-Glc*p*-(1→
22.474	2,3,4-Me_3_-Man*p*	43,71,87,99,101,117,129,159,161	0.282	→6)-Man*p*-(1→
24.166	2,3,4-Me_3_-Gal*p*	43,87,99,101,117,129,161,189,233	0.052	→6)-Gal*p*-(1→
28.430	2,4-Me_2_-Man*p*	43,87,117,129,159,189,233	0.099	→3,6)-Man*p*-(1→
29.438	2,4-Me_2_-Gal*p*	43,87,117,129,159,189,233	0.066	→3,6)-Gal*p*-(1→

**Table 3:** ^13^C-NMR and ^1^H-NMR spectral assignments of MSEPS.

	Glycosyl Residues		H1/C1	H2/C2	H3/C3	H4/C4	H5/C5	H6a/C6	H6b
A	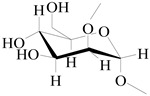	→2)-α-d-Man*p*-(1→	5.21	4.00	3.88	3.60	3.70	3.69	3.81
			101.75	80.21	71.32	68.19	74.50	62.30	
B	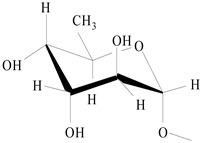	α-l-Fuc*p*-(1→	5.16	3.52	nd	nd	nd	1.14	
			101.96	73.23	nd	nd	nd	15.31	
C	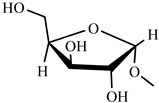	α-l-Ara*f*-(1→	5.01	4.13	3.83	3.91	3.69		
			108.77	82.70	77.80	85.10	62.33		
D	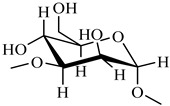	→3)-α-d-Man*p*-(1→	4.99	3.74	3.78	4.24	3.91	3.73	
			95.78	69.36	80.50	68.33	71.00	62.10	
E	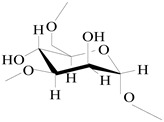	→3,6)-α-d-Man*p*-(1→	4.87	4.09	3.82	3.65	3.73	3.68	3.87
			100.84	68.18	79.46	72.70	74.64	66.91	
F	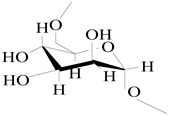	→6)-α-d-Man*p*-(1→	4.82	3.91	3.75	3.75	3.68	3.92	3.89
			100.80	71.37	71.87	67.89	74.50	66.82	
G	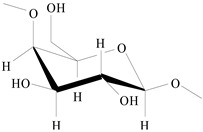	→4)-β-d-Gal*p*-(1→	4.57	3.61	3.69	4.10	3.63	3.73	
			105.80	73.21	74.84	79.66	76.01	62.1	
H	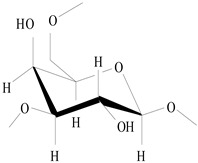	→3,6)-β-d-Gal*p*-(1→	4.46	3.57	3.68	4.05	3.87	3.96	3.86
			104.69	71.31	81.50	69.82	74.81	70.76	
I	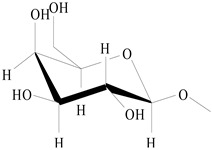	β-d-Gal*p*-(1→	4.25	3.28	3.46	3.21	3.60	3.54	3.41
			104.48	74.66	76.62	83.40	77.36	64.00	

nd: not detected.

## Data Availability

The data presented in this study are available in the article.
